# Informed Consent in Paediatric Telemedicine: Challenge or Opportunity? A Scoping Review

**DOI:** 10.3390/healthcare11101430

**Published:** 2023-05-15

**Authors:** Giovanna Ricci, Filippo Gibelli, Paolo Bailo, Anna Maria Caraffa, Giulio Nittari, Ascanio Sirignano

**Affiliations:** 1 Section of Legal Medicine, School of Law, University of Camerino, 62032 Camerino, Italy; 2 Telemedicine and Telepharmacy Centre, School of Medicinal and Health Products Sciences, University of Camerino, 62032 Camerino, Italy

**Keywords:** telemedicine, paediatrics, paediatric teleconsultation, informed consent

## Abstract

The fundamental importance of informed consent as a prerequisite for the lawfulness of the medical act is an indisputable cornerstone of clinical practice. However, the provision of effective information and the collection of informed consent presents important critical issues in the underage patient, even considering that in general terms he or she does not have the power to directly express consent, which must be provided by parents or legal guardians. These critical issues are amplified in the context of telemedicine. The present study aims, through a scoping review of the literature of the past 10 years, to outline the operational practices adopted in the collection of informed consent from children in the context of telemedicine and to identify solutions devised to address the critical issues related to the provision of adequate information to the child in this particular care setting. The results of the research show that the activity of delivering adequate information to the child, itself complex, is made even more complex by the particular setting of telemedicine, which, however, could be effectively exploited to facilitate communication with the child patient.

## 1. Introduction

Informed consent given by the patient represents a necessary prerequisite for any medical activity, whether diagnostic or therapeutic. Providing consent to the medical act allows the patient to exercise the right to self-determination. The fundamental importance of consent as an essential element of medical activity was codified for the first time in the Nuremberg Code of 1947, a set of ethical principles for human experimentation drafted at the end of the trials against Nazi hierarchs.

The need to obtain the consent of the person undergoing experimentation was then reaffirmed in 1964 with the Declaration of Helsinki, one of the key documents of ethics in clinical research. The concept of informed consent as an essential element for the implementation of any medical act was officially recognised in 1997 with the Convention for the Protection of Human Rights and Dignity of the Human Being with regard to the Application of Biology and Medicine, signed in Oviedo, Spain.

The Oviedo Convention also deals with the issue of the validity of the informed consent of a minor, establishing that a medical act to be performed on a person who has not yet reached the age of majority may only be performed once the consent of his or her representative, an authority, or a person or body provided for by law has been obtained. The document specifies that due consideration should always be given to the views of the minor, in relation to his or her age and level of maturity. Therefore, the principle is established according to which the minor’s ability to understand and make decisions must be enhanced as much as possible. According to the most widespread legal orientations, three age brackets are identifiable in relation to the right of minors to express consent to the medical act: children under the age of 6–7 may not express any form of consent; children aged between 7 and 13 must be involved to some extent in the decision-making process; and the opinion of children over the age of 14 years should be considered to be almost like an adult’s opinion (although the provision of consent remains a parental prerogative).

As far as the European context is concerned, in the United Kingdom, the principle of “Gillick competence” allows minors to consent to medical treatment if they demonstrate sufficient understanding and intelligence. This approach is case-specific and does not set a specific age limit. In France, the age of medical consent is 16 years. However, minors can participate in the decision-making process if they are deemed capable of understanding the implications of a medical procedure. In cases where the minor is under 16, parental consent is required alongside the minor’s consent, reflecting the “double consent” rule [1]. In Germany, minors aged 14 and above can provide informed consent for medical treatment, provided they understand the consequences. For those under 14, parental consent is necessary. In Spain, minors aged 16 and older can independently consent to medical procedures. The double consent rule is applied to children aged 12–15, where both minor and parent must consent. In the Netherlands, minors aged 12 and above can provide informed consent if they have the capacity to understand the medical procedure. For those under 16, the double consent rule applies. In Sweden, the age of medical consent is 15 years. However, minors under 15 may consent alongside their parents, again following the double consent rule. In Belgium, minors aged 15 and above can consent to medical treatment independently. For minors below 15, double consent is required [2]. The double consent rule is applied in the majority of the countries discussed for minors between the ages of 12 and 15. This ensures that the minor’s autonomy is respected while also considering the parents’ responsibility in medical decision-making. The rule acknowledges that minors within this age range may possess varying levels of maturity and understanding and, therefore, require additional support from their parents. By applying the double consent rule, these countries strike a balance between safeguarding the interests of minors and acknowledging their developing autonomy [3].

The issue of informed consent of minors is particularly interesting especially if considered in relation to the technological evolution of medicine and progress in the field of communication. Today, a considerable number of medical services are provided in the absence of direct physical contact between physician and patient [4,5,6]. This is telemedicine, a system of interactive multimedia communication that is now a concrete reality of the health system and which is increasingly widespread. The healthcare services provided through telemedicine are, to all intents and purposes, regular healthcare services and as such are subject to the acquisition of informed consent from the patient. The telematic modality poses obvious logistical difficulties for the healthcare provider in acquiring informed consent (especially in written form) [7,8].

The healthcare professional essentially has three options when documenting the acquisition of informed consent in telemedicine services. These options are video recording, audio recording, and requiring the patient to fill out pre-printed forms sent by e-mail. In the case of video recording and audio recording, the acquisition of consent is preceded by sending through an electronic system (by e-mail or through instant messaging applications) a detailed information report to the patient [9]. The patient’s willingness to undergo the treatment or diagnostic investigation is then recorded (by video in the case of videoconferencing or by simple audio in the case of telemedicine services that do not involve the use of a video camera). Regarding the third option, the Agency for Healthcare Research and Quality developed a detailed operational guide explaining how to acquire consent through the development of a special form sent in advance by email to the patient [10].

When the issue of obtaining informed consent in telemedicine is dropped into the paediatric context, complications increase. Recent advances in technology and the evolving landscape of healthcare delivery have indeed paved the way for telemedicine to play an increasingly significant role in paediatric care. Telemedicine has shown promise in various paediatric subspecialties, such as telepsychiatry, teleradiology, and telecardiology, among others [11]. Furthermore, telemedicine has proven to be beneficial in addressing healthcare disparities by increasing accessibility to care for rural and underserved populations [12]. As the technology continues to advance and adoption barriers are addressed, it is reasonable to envision a future where telemedicine becomes an integral part of paediatric care, and for this reason the subject of informed consent in paediatric telemedicine is gaining significant prominence today.

A recent German study attempted to define the state of the art in relation to computerised acquisition of informed consent for surgical interventions, with particular reference to paediatric surgery. The researchers sent a web-based questionnaire regarding the informed consent process to members of the European Society of Anaesthesia and Intensive Care Medicine from 47 European countries (42,433 recipients/930 responses). Six questions in the questionnaire specifically concerned paediatric telemedicine. According to the survey results, the majority of respondents, 70.2%, believed that obtaining informed consent through the internet in a regular setting was not feasible. Additionally, 67.3% of the participants were unsure if such practices aligned with legal regulations. In the field of paediatric anaesthesia, 77.6% of respondents felt that obtaining informed consent from only one parent was sufficient for simple interventions, while 63.8% felt the same for complex interventions. Around half of the respondents believed that verifying the parents’ identity was necessary, but only 29.9% reported actually requesting it in their clinical routine [13].

## 2. Materials and Methods

We conducted a scoping review based on the approach suggested by Arksey and O’Malley [14], which consists of 5 steps:

Step 1. Identifying the research questions.
Step 2. Identifying relevant studies.
Step 3. Selecting studies.
Step 4. Charting the data.
Step 5. Collating, summarising, and reporting the results.

In the present paper, we used the methodological approach of the scoping review in order to provide a comprehensive overview of the critical issues related to the provision of information to the child in telemedicine and the collection of informed consent. We conducted this research not only to outline the state of the art on the topic as it emerged from the study of the scientific literature, but also to see whether the special care context of telemedicine can be exploited to improve the quality of information provision to the child patient.

### 2.1. Identifying the Research Questions

We developed the questions on which this study is based, in accordance with the population/concept/context (PCC) framework suggested by the Joanna Briggs Institute (JBI) [15]. We opted to use the JBI methodology to develop the review questions, as the PCC framework it employs is widely acknowledged for its ability to effectively address the specific requirements of a scoping review by taking a broader approach than a systematic review [16,17]. Thus, we determined that utilizing the PCC approach would be the most efficient method for formulating the primary and secondary questions of the review, acknowledging the necessity for them to encompass a wide range of topics.

The primary review question was as follows:

(1) What are the major critical issues encountered in providing medical information to minor patients through telemedicine services and collecting their informed consent?

Two subquestions then spontaneously emerged:

(2) If properly utilised, could telemedicine services facilitate adequate transmission of medical treatment information?

(3) Is it possible to propose an operational mode of delivery of medical information to the paediatric patient in telemedicine and consent collection?

### 2.2. Identifying Relevant Studies

#### 2.2.1. Databases

We used three databases in this review based on our topic: PubMed, Scopus, and Web of Science. We voluntarily omitted databases searching for grey literature for two reasons: due to the highly specialised and technical nature of the topic, we faced challenges in locating narratives, commentaries, reports, and essays that directly addressed the subject matter. Additionally, to ensure the highest level of scientific rigor in our research, we decided to exclude texts without scrupulously documented scientific validity.

#### 2.2.2. Inclusion Criteria. The Application of the PCC Framework

Regarding the “population” field, we chose to consider the paediatric population as a matter of course, and thus defined it as those under the age of 18, 18 being the required age of majority in most European countries. Given that children under the age of 7 are generally not mature enough to be involved in the decision-making process, only children between the ages of 7 and 18 are considered. Since minors are involved and the final consent is usually given by parents, we also incorporated the concept of “parents” in the search to ensure the relevancy of the results. In the “concept” field, we included two concepts: the concept of acquiring informed consent and the concept of communication (referring to communication between parents and children). The context is, of course, telemedicine. For each keyword, we identified several medical subject headings (MeSH) and synonyms to be used as alternative keywords. Table 1 illustrates the application of the PCC framework to the scoping review questions.

#### 2.2.3. Search Strategy

In accordance with the methodological approach suggested by the Joanna Briggs Institute [18], the first step consisted of a preliminary search within the Pubmed database. For each PCC element, we introduced the relevant MeSH and keywords, and then we joined the lines related to them to obtain an overall set line for that specific PCC element, combining them with the “OR” Boolean operator. Finally, we combined all overall set lines with the “AND” Boolean operator, to find the results that addressed all our PCC elements. We did not set limits in relation to study design. Regarding the time window, we restricted the search to studies reported in the last 10 years (between 1 January 2013 and 1 January 2023). We considered only articles in English. Below is the search string entered:

*((“informed consent”[MeSH Terms] OR “informed consent”[All Fields])) AND ((“telemedicine”[MeSH Terms] OR “telemedicine”[All Fields] OR “remote consultation”[All Fields] OR “virtual consultation”[All Fields])) AND ((“paediatrics”[MeSH Terms] OR “paediatrics”[All Fields] OR “child”[All Fields] OR “adolescent”[MeSH Terms] OR “adolescent”[All Fields])) AND ((“parents”[MeSH Terms] OR “parents”[All Fields] OR “communication”[MeSH Terms] OR “communication”[All Fields])) AND “English”[Language] AND (“2013/01/01”[Date—Create]: “2023/01/31”[Date—Create])*

We obtained 47 resulting articles. We then applied the same methodological approach—making the necessary adjustments to keywords and MeSHs—on the databases Scopus (30 resulting articles) and Web of Science (4 resulting articles). The following are the strings used for Scopus and Web of Science:

SCOPUS

*(TITLE-ABS-KEY(“informed consent”) AND TITLE-ABS-KEY(“telemedicine” OR “remote consultation” OR “virtual consultation”) AND TITLE-ABS-KEY(“paediatrics” OR “child” OR “adolescent”) AND TITLE-ABS-KEY(“parents” OR “communication”) AND LANGUAGE (English) AND PUBYEAR > 2012 AND PUBYEAR < 2024)*

WEB OF SCIENCE

*(TS = (“informed consent”) AND TS = (“telemedicine” OR “remote consultation” OR “virtual consultation”) AND TS = (“paediatrics” OR “child” OR “adolescent”) AND TS = (“parents” OR “communication”) AND LANGUAGE: (English) AND PY = 2013 − 2023)*

Overall, we found 81 articles using the above search terms and databases.

We completed the last search on 14 February 2023.

### 2.3. Identifying Relevant Studies

Once we completed the bibliographic collection phase, we entered the 81 articles obtained from the three databases into EndNote software. The initial stage involved using an automated software tool to identify and remove duplicate articles (n = 18). At the end of the initial skimming procedure, we obtained a library of 63 articles. Afterwards, we employed an EndNote tool to perform an initial screening phase to exclude articles that were not relevant to the review’s objective. As a result, 15 articles were excluded at the end of the initial screening because they were clearly unrelated to the research topic. Of the remaining 48 articles, the title and abstract were read: 11 were excluded because, although they related to technological tools in healthcare, they did not appear to deal specifically with the field of telemedicine, and 23 were excluded because they did not relate to the paediatric population. The remaining 14 articles, the full text of all of which could be found, were read in full. They were all deemed fit for the purpose of the review.

### 2.4. Charting the Data

In order to have the necessary data to answer the review questions, we employed a data charting form using the spreadsheet program Excel. We decided to extract the following data from the selected individual articles:

1. Author(s)
2. Title
3. Year of publication
4. Geographical context
5. Aim of the study
6. Patients’ age
7. Consent collection method

The title of the article facilitated the ready identification of the central focus of the research, while the year of publication and the geographic context provided useful indications of the technological and geographic–cultural context underpinning the research. The age of the children was crucial in understanding the degree of maturity of the patients and thus in understanding which modes of acquiring consent could be developed.

### 2.5. Collating, Summarising, and Reporting the Results

**Figure 1:**
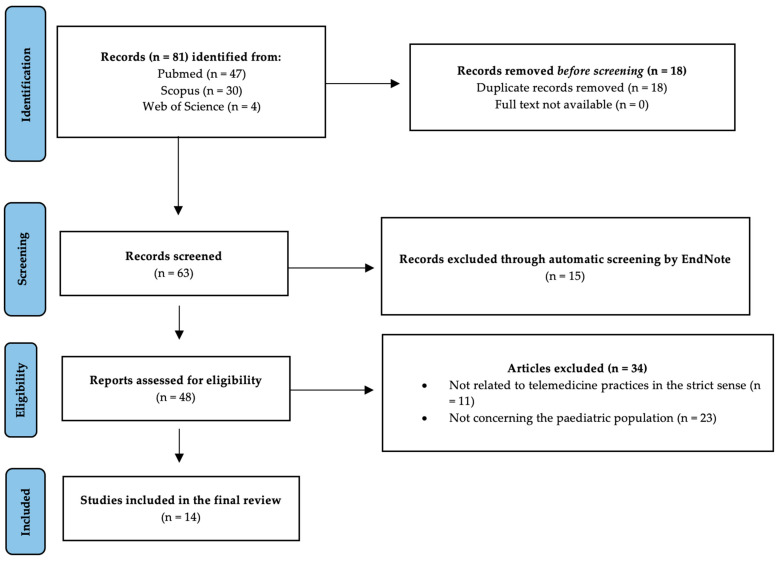
PRISMA-ScR (preferred reporting for systematic reviews and meta-analysis extension for scoping reviews) flow diagram for study selection.

We reported the results of the research in two different ways: a flow chart illustrating the main stages of the research that led to the results (Figure 1) and a summary table showing the descriptive elements used in the data charting (Table 2).

## 3. Discussion

It is noteworthy that in all of the articles obtained, most of which were clinical trials, consent was acquired in the traditional manner before providing telemedicine services. This is likely due to the challenges presented by teleconsultation in terms of providing adequate and complete information and obtaining consent (which is typically obtained through a signature on a sheet). Therefore, it is commonly assumed that effective information activities can only be achieved through direct physical contact between the doctor or investigator and the minor patient or parent/guardian.

Before discussing the context of information provision and consent delivery (physical or virtual), it is important to consider whether the content of information provided in telemedicine services is analogous to that of traditional health services. The answer is no. When it comes to telemedicine services, it is crucial to provide more information compared to what is typically provided in a conventional medical service. Specifically, three aspects require additional attention: the technical limitations of the telemedicine service, which cannot match the traditional service in many respects; the possibility of interference due to poor quality of the internet signal; and the inability of the doctor to directly intervene in the event of need. These are the three aspects whose importance was brought up in the National Guidelines on Telemedicine issued by the Italian Ministry of Health in July 2012 [33].

In considering the context of information provision (physical or virtual), significant challenges arise when obtaining consent in a video consultation/visit scenario, where the health professional is situated on one side of the virtual table, and the child is accompanied by the parent/guardian on the other side.

Firstly, how to obtain a virtual signature? Given the widespread use of digital identification systems (in Italy the SPID (Public Digital Identity System) and the electronic identity card and in Europe the EIDAS (Electronic IDentification and Trust Services) and the FIDO (Fast IDentity Online)), there do not seem to be any real difficulties in obtaining the patient’s signature. If there are difficulties in using these systems, one can always resort to signing on paper, then scanning the sheet and sending the scanned file by e-mail.

The second critical issue is related to ascertaining the authenticity of the parent or guardian’s identity, thereby ensuring that the person claiming to be them is, in fact, genuinely who they purport to be. Verifying the identity of the parent or guardian in a telemedicine service involving a minor is essential to ensure patient safety and the legality of medical practice. Simply bringing the identity document close to the webcam may prove insufficient, as it is impossible to physically check the authenticity of the document. The parent/guardian could be asked to bring a copy of the child’s birth certificate close to the webcam, along with their identity card. Regardless, the best way to verify that the parent/guardian really is whom they claim to be is through the use of an authentication technology solution, such as facial biometrics or fingerprint-based identification.

A third aspect that makes the acquisition of informed consent in the context of paediatric telemedicine services particularly complex is the difficulty of protecting the privacy of the child patient. Generally, in traditional medicine services, since consent is given by the parent/guardian, the information interview with the doctor involves both the minor patient and the parent/guardian. However, the interview between a minor patient and the physician may take place without the presence of the parent or guardian in cases where the minor has reached an age at which he or she is considered mature enough and capable of making informed decisions about his or her own health, and if the doctor believes that the presence of the parent or guardian may negatively influence communication with the minor. Thus, the problem of the protection of the minor’s privacy intersects with that of possible interference by the parent/guardian, whose presence may influence the minor’s choice. In the context of a traditional health service, should it be necessary to talk to the minor alone, it would be sufficient to require the parent/guardian to leave the room. In the case of a telemedicine service, it is much more complex to ensure that the parent/guardian does not actually participate in the videoconference conversation. The doctor could ask the parent/guardian to leave the room in which the computer is located, but this does not ensure that the parent/guardian is actually prevented from hearing the conversation between the doctor and the child. One solution could be to ask the minor patient to use a device other than the one used by the parent or guardian for the video call, so that the doctor can have a private conversation with the minor. Another solution could be to use encryption technologies to protect communications between the doctor and the minor patient and to limit access to the patient’s information to authorised personnel only.

Beyond the critical issues described above, for which solutions can easily be found, it should not be forgotten that telemedicine services can potentially represent a tool that can make computerised activity preceding the provision of consent even more effective and thus able to make consent even more “informed”. In a teleconsultation, the use of a video platform and other technologies can offer unique advantages for communication between the doctor and the patient. For example, during a videoconference, the doctor can share images, charts, and other visual information in real time. This can help the patient better understand the information the doctor is providing, as visual information is often easier to comprehend than verbal information. Additionally, during a videoconference, the physician can use screen-sharing tools to show the patient websites, documents, and other information relevant to their health. This way, the patient can access information in real time during the consultation, rather than having to search for information on their own after the visit. Finally, during a teleconsultation, patients may feel more in control of the conversation and more involved in their care, as they are able to ask questions more directly and get detailed answers from the doctor. This can lead to greater understanding and adherence to the doctor’s recommendations, thus improving the quality of healthcare. All these aspects are even more relevant when considered with reference to paediatric telemedicine, given that children are certainly more attracted to multimedia content and are therefore more inclined to take in information conveyed through animations/images.

## 4. Conclusions

As a result of the literature review, including by examining the most relevant scientific production on the subject, the initial questions can be answered as follows.

*(1) What are the major critical issues encountered in providing medical information to minor patients through telemedicine services and collecting their informed consent?*

The main critical issues are of a technical–operational nature and related to the methods of identifying the child and parent, the ability to interact with them separately, and the methods of obtaining consent. However, these are problems for which simple solutions can be identified, and once identified, the advantages that virtual communication can bring can be highlighted.

Beyond the operational challenges, there are also legal complexities that must be addressed. Informed consent in paediatrics has always been complex due to the involvement of both the child and their legal guardian. Telemedicine adds another layer of complexity, as issues such as privacy, data security, and jurisdictional concerns come into play. It is essential to establish clear guidelines and protocols to ensure that informed consent in telemedicine is in line with ethical principles and legal requirements. To mitigate potential legal repercussions, healthcare providers must ensure that consent is obtained in a manner that respects patient autonomy and adheres to established standards of care. This may involve providing comprehensive information about the telemedicine process, addressing privacy concerns, and ensuring that patients and their guardians understand the potential risks and benefits of telemedicine. Additionally, healthcare providers should stay informed about the evolving legal landscape surrounding telemedicine, as regulations and laws may change over time. By addressing both operational and legal challenges, the full potential of telemedicine in paediatrics can be realised while maintaining the highest standards of patient care.

*(2) If properly utilised, could telemedicine services facilitate adequate transmission of medical treatment information?*

If used intelligently, telemedicine services can undoubtedly facilitate better transmission of information on medical care. This can be achieved above all through the ethical use of the numerous functionalities that a virtual communication system makes available. To date, it appears that these advantageous aspects have not yet been fully exploited, probably because we are still in the first phase of the cultural evolution that accompanies an informed use of telemedicine.

*(3) Is it possible to propose an operational mode of delivery of medical information to the paediatric patient in telemedicine and consent collection?*

We believe it is possible. In fact, we have developed a possible model of information provision and consent collection in telemedicine. This model is designed for consent collected directly in virtual mode and is structured as follows. The information activity must include separate sessions: one session for children and one for parents. The assurance that the sessions are truly separate, with no parental influence on the children, can take place, for example, via different devices or with encryption systems. In the session with the parent/guardian, the information can be delivered in the traditional way, i.e., verbally or by means of written forms. In the session with the child, the information must be tailored to the child’s age and linguistic, cognitive, and comprehension skills and must make use of graphic, interactive, or multimedia communication tools. At the end of the separate sessions, a moment of private discussion between the parent/guardian and the child must be guaranteed, at the end of which consent will be collected.

This scoping review has several limitations that should be acknowledged when interpreting the findings. Firstly, the review was limited to articles retrieved from Scopus, PubMed, and Web of Science databases. This may have led to the exclusion of relevant studies published in other databases or grey literature sources. Moreover, the search strategy might have unintentionally missed some pertinent articles due to the use of specific keywords. Secondly, the rapidly evolving nature of telemedicine technology and regulations presents a challenge in drawing definitive conclusions. As the field continues to develop, new tools and practices may emerge that address some of the identified critical issues, rendering some findings of this review less relevant. In this context, it is essential to keep in mind that the proposed operational mode of information delivery and consent collection may require continuous adaptation to remain effective. Thirdly, the review did not explicitly consider the potential impact of cultural, linguistic, and socioeconomic differences on the provision of medical information and collection of informed consent in paediatric telemedicine. These factors can significantly influence the way telemedicine services are utilised and perceived by different populations, and addressing these issues may be crucial for the successful implementation of telemedicine in diverse settings. Lastly, the proposed model for information provision and consent collection has not been empirically tested or validated in real-world settings. While the model is theoretically sound and considers several important aspects of telemedicine in paediatrics, its practical feasibility and effectiveness remain to be determined through rigorous research and evaluation. In light of these limitations, further research is required to expand our understanding of informed consent in paediatric telemedicine and to refine the proposed model to ensure its applicability across diverse contexts and populations.

## Figures and Tables

**Table 1:** The PCC framework (inclusion criteria).

	Main Concept	Alternate Keywords	Subject Headings (MeSH)
Population	Children	Paediatrics; child	Paediatrics; Adolescent
	Parents	/	Parents
Concept	Acquiring informed consent	Informed consent	Informed Consent
	Communication (between parents and children)	/	Communication
Context	Telemedicine	Remote consultation; virtual consultation	Telemedicine

**Table 2:** The 14 studies included in the review.

Reference	Title	Year	Geographical Context	Aim of the Study	Patients’ Age	Consent Collection Method
Yager et al. [19]	Reliability of circulatory and neurologic examination by telemedicine in a Pediatric Intensive Care Unit	2014	USA	Randomised prospective study in a 14-bed PICU in a tertiary care, academic-affiliated institution having the objective of comparing telemedicine with face-to-face assessment of patients undergoing circulatory or neurological examinations	Between 2 months and 19 years	Informed consent was obtained from all patients (or parents/guardians) before participation in the study after reading and understanding a written information sheet containing objectives, risks, and benefits
Hardy et al. [20]	The added value of a mobile application of Community Case Management on referral, re-consultation and hospitalization rates of children aged under 5 years in two districts in Northern Malawi: study protocol for a pragmatic, stepped-wedge cluster-randomised controlled trial	2017	Malawi	Stepped-wedge cluster-randomised trial with a pragmatic approach conducted to evaluate the impact of the SL eCCM App (Supporting LIFE electronic Community Case Management Application) on rates of urgent referral, re-consultation, and hospitalization of children within 7 days of the index visit	Between 2 months and 5 years	Given the age of the children, consent was obtained from caregivers prior to the start of the study after reading a written consent form
Ramelet et al. [21]	Impact of a nurse led telephone intervention on satisfaction and health outcomes of children with inflammatory rheumatic diseases and their families: a crossover randomised clinical trial	2017	Switzerland	Multicentre, randomised, longitudinal crossover study conducted in paediatric outpatients with newly diagnosed inflammatory rheumatic diseases in order to compare telenursing services with traditional medical care services	Under 16 years	Consent was obtained directly from children (if over 11 years old) or from parents through a written form before the trial began
Rhodes et al. [22]	A telephone intervention to achieve differentiation in dietary intake: a randomised trial in paediatric primary care	2017	USA	Randomised trial aimed at evaluating whether dietary advice based on two healthy nutrition programs can be effectively provided to families of obese children by telephone	Between 5 and 10 years	Consent was obtained directly from children (if over 7 years old) or from parents after an information activity delivered by telephone
Nyström et al. [23]	A 12-month follow-up of a mobile-based (mHealth) obesity prevention intervention in pre-school children: the MINISTOP randomised controlled trial	2018	Sweden	Two-arm parallel randomised controlled trial aimed at testing the effectiveness of an app to combat obesity in preschool children	4.5 years	Consent was obtained from parents
Franke et al. [24]	A mobile phone based tool to identify symptoms of common childhood diseases in Ghana: development and evaluation of the integrated clinical algorithm in a cross-sectional study	2018	Ghana	Study aimed at the development and evaluation of an algorithm-based diagnostic tool, applicable on mobile phones, to support parents/guardians in providing appropriate care to sick children	Between 1 month and 15 years	Written informed consent was obtained from the parents/guardians before the start of the study
Sgandurra et al. [25]	Early intervention at home in infants with congenital brain lesion with CareToy revised: a RCT protocol	2018	Italy	Randomised controlled trial aiming to evaluate the efficacy of CT-R (a medical device that delivers an early, intensive, customised, intervention program, carried out at home by parents but remotely managed by expert and trained clinicians) compared to Infant Massage (IM) intervention in a sample of infants at high-risk for cerebral palsy,	Preterm or full-term infants with brain lesions, in first year of life	Parents signed two informed consent forms (one for each phase of the study) after receiving detailed information in both written and oral form
Simone et al. [26]	Computer-assisted rehabilitation of attention in pediatric multiple sclerosis and ADHD patients: a pilot trial	2018	Italy	Pilot double-blind randomised controlled trial to evaluate the efficacy of a home-based computerised-program for retraining attention in two cohorts of POMS (Paediatric Onset Multiple Sclerosis) and ADHD (Attention Deficit/Hyperactivity Disorder) patients	Under 18 years	Informed consent form was signed by the parents of the participants
Strickler et al. [27]	Contribution of the use of basic telemedicine tools to the care of children and adolescents with juvenile idiopathic arthritis at the Puerto Montt Hospital, Chile	2018	Chile	Retrospective study consisting of a review of the medical records of children over 14 years of age with juvenile idiopathic arthritis undergoing clinical monitoring via a mixed system (face-to-face and telemedicine visits)	Under 18 years	Informed consent to the study was provided directly by the patients
Ramkumar et al. [28]	Implementation and evaluation of a rural community-based pediatric hearing screening program integrating in-person and tele-diagnostic auditory brainstem response (ABR)	2019	India	Evaluation of the effectiveness of a paediatric hearing screening programme by integrating two diagnostic ABR (Auditory Brainstem Response) test models: one using a telemedicine approach and the other a traditional test	Under 5 years	Written and verbal informed consent was obtained from parents
Browne et al. [29]	Mobile Health Apps in Pediatric Obesity Treatment: Process Outcomes From a Feasibility Study of a Multicomponent Intervention	2020	Republic of Ireland	Evaluation of the usability of 2 m-Health (mobile health) applications as an adjunct to traditional treatment for obesity	Between 9 and 16 years	Informed consent given after reading information leaflet by children and parents
Kobel et al. [30]	Accuracy of the Apple Watch iECG in Children With and Without Congenital Heart Disease	2022	Germany	Evaluation of the agreement of measured values of rate, interval, and amplitude with those obtained by a diagnostic quality ECG recording to an Apple Watch iECG in children with and without congenital heart disease	Between 0 and 16 years	Consent obtained from parents
Smith et al. [31]	Therapist-supported online cognitive therapy for post-traumatic stress disorder (PTSD) in young people: Protocol for an early-stage, parallel-group, randomised controlled study (OPTYC trial)	2022	UK	Early-stage trial aimed at gathering data on feasibility, acceptability, and initial indications of clinical efficacy of internet-delivered cognitive therapy for post-traumatic stress disorder in young people	Between 12 and 17 years	For participants under 16 years of age, informed consent was provided by the parents/guardians after asking for the patients’ consent. Participants aged 16 years and older gave consent independently, without parental involvement
Sonney et al. [32]	Improving Asthma Care Together (IMPACT) mobile health intervention for school-age children with asthma and their parents: a pilot randomised controlled trial study protocol	2022	USA	Pilot randomised controlled trial aimed at determining the feasibility, acceptability, and preliminary efficacy of the IMPACT intervention, a novel shared management system composed of a mobile health (mHealth) application, symptom watch, and tailored health intervention that pairs parent and child together as an asthma management team	Between 7 and 11 years	Both study participants (children) and parents were asked to give consent. Parents accessed an electronic information form with electronic signature, and children accessed a short video explaining the purpose and modalities of the study. The study team then contacted the participants to answer any questions and discuss consent.

## Data Availability

No applicable.
